# Variability in monocular and binocular fixation during standard automated perimetry

**DOI:** 10.1371/journal.pone.0207517

**Published:** 2018-11-21

**Authors:** Kazunori Hirasawa, Kaoru Kobayashi, Asuka Shibamoto, Houmi Tobari, Yuki Fukuda, Nobuyuki Shoji

**Affiliations:** 1 Department of Ophthalmology, School of Medicine, Kitasato University, Kanagawa, Japan; 2 Moorfields Eye Hospital NHS Foundation Trust and University College London, Institute of Ophthalmology, London, United Kingdom; 3 Department of Ophthalmology, Keio University School of Medicine, Tokyo, Japan; 4 Discipline of Orthoptics, Graduate School of Health, University of Technology Sydney, Sydney NSW, Australia; 5 Department of Ophthalmology, The Jikei University School of Medicine, Tokyo, Japan; University of Tübingen, GERMANY

## Abstract

The aim of this cross-sectional study was to use standard automated perimetry to compare fixation variability among the dominant eye fixation, non-dominant eye fixation, and binocular fixation conditions. Thirty-five eyes of 35 healthy young participants underwent standard automated perimetry (Humphrey 24–2 SITA-Standard) in dominant eye fixation, non-dominant eye fixation, and binocular fixation conditions. Fixation variability during foveal threshold and visual field measurement, which was recorded using a wearable eye-tracking glass and calculated using the bivariate contour ellipse area (deg^2^), was compared among the three fixation conditions. Further, the association of bivariate contour ellipse area with ocular position and fusional amplitude during binocular fixation was analysed. There were no significant differences in bivariate contour ellipse area during foveal threshold measurement among the dominant eye fixation (1.75 deg^2^), non-dominant eye fixation (1.45 deg^2^), and binocular fixation (1.62 deg^2^) conditions. In contrast, the bivariate contour ellipse area during visual field measurement in binocular fixation (2.85 deg^2^) was significantly lower than the bivariate contour ellipse area in dominant eye fixation (4.62 deg^2^; p = 0.0227) and non-dominant eye fixation (5.24 deg^2^; p = 0.0006) conditions. There was no significant difference in bivariate contour ellipse area during visual field measurement between dominant eye fixation and non-dominant eye fixation conditions. There was no significant correlation between bivariate contour ellipse area and either ocular position or fusional amplitude during both foveal threshold and visual field measurements. Thus, fixation variability might be improved in binocular fixation conditions during a long-duration test, such as visual field measurement.

## Introduction

Perimetry is the systematic measurement of visual field function performed during central fixation without eye movement. Previous studies have reported that fixation variability affects the test–retest variability of retinal sensitivity in healthy participants[1] and patients with glaucoma[2]; additionally, it affects the detection of scotoma.[3] Therefore, fixation monitoring during visual field measurement is very important for accurate test results.

Several methods have been used for fixation monitoring during standard automated perimetry (SAP). The Humphrey Field Analyzer (HFA; Carl Zeiss Meditech, Dublin, CA) employs the gaze-tracking system and the Heijl–Krakau blind-spot monitoring method,[4] while the Octopus perimeter (Haag-Streit, Koeniz, Switzerland) employs a video monitor with a display and an automatic eye-tracking system. The MP-1/MP-3 (NIDEK, Aichi, Japan)[5, 6] and Compass (CenterVue, Padova, Italy)[7, 8] perimeters employ the fundus-tracking approach. In addition, imo, a head-mounted perimeter (CREWT, Tokyo Japan) employs the pupil-tracking method.[9]

Many studies have performed quantitative evaluation of fixation variability during static perimetry.[10–26] Importantly, fixation variability during static perimetry is reported to be relatively high among patients with glaucoma,[10, 18, 20] age-related macular degeneration,[20–23, 25, 27] diabetic maculopathy,[14, 20] and macular holes.[24] Additionally, in healthy participants, fixation variability has been reported to decrease when using a smaller fixation target size.[1, 19, 27] However, most previous studies have evaluated fixation variability exclusively in monocular fixation conditions.

In the past, visual field measurement was generally performed separately in each eye, because the visual field of one eye compensates for that of the other. However, this method of measurement was reported to suffer from the blank-out phenomenon.[28, 29] A previous study reported on the value of visual field measurement in each eye with binocular viewing using a head-mounted perimeter, imo, which can randomly present stimuli to either eye without occlusion and without examinee awareness of which eye is being tested (the binocular random single-eye test); this measurement was useful in healthy paediatric subjects and in young subjects with psychosomatic visual field abnormalities.[30] Previous studies of fixation variability during monocular and binocular viewing, in patients with age-related macular degeneration, reported that fixation variability in the worse eye was 84–100% better in the binocular condition than it was in the monocular condition.[31] Additionally, the advantage of fusion is introduced in the binocular condition, suggesting that this condition might be advantageous for visual field measurement, compared with monocular fixation. However, there are no published reports utilizing SAP measurement to compare fixation variability among dominant eye fixation (DEF), non-dominant eye fixation (N-DEF), and binocular fixation (BF) conditions.

This study aimed to compare fixation variability during SAP between monocular and binocular fixation conditions, and to evaluate the association of fixation variability with ocular position and fusional amplitude during BF.

## Methods

This study followed the tenets of the Declaration of Helsinki and was approved by the ethics committee of Kitasato University School of Allied Health Science (No. 2016–05). All participants provided written informed consent. This study was conducted between May and November 2016 and has been registered in the UMIN Clinical Trials Registry (http://www.umin.ac.jp/) under the unique trial number UMIN000022381 (date of registration: 05/20/2016).

This cross-sectional study included 35 healthy student volunteers enrolled in the Orthoptic and Visual Science course at Kitasato University, who had at least three previous SAP experiences within 3 months before enrolment in this study. All participants underwent comprehensive ophthalmic examination, including non-cycloplegic refraction testing, visual acuity testing at 5 m with a Landolt ring chart, intraocular pressure measurement, ocular axial length measurement, slit-lamp and fundus examination by a glaucoma specialist (NS), ocular position test, and fusional amplitudes test. Participants were included in this study if they exhibited a corrected visual acuity of 20/20 or better, intraocular pressure of ≤21 mmHg, cylindrical power of ≤1.50 dioptre (D), as well as a normal optic-disc appearance, no ophthalmic diseases that could affect the results of the visual field test, and no manifest or intermittent strabismus.

SAP was performed using the HFA 24–2 Swedish Interactive Threshold Algorithm. Since the participants were all young, refractive error was corrected for far distance using disposable soft contact lens (1-Day Acuvue, Johnson & Johnson Vision Care, Inc., New Brunswick, NJ). During BF measurement, the chin rest was moved to the extreme right, and the participants rested their chin on the left chin rest. The middle of the right and left eyes was positioned at the centre of the monitor. In addition, fixation monitoring of the Heijl–Krakau blind spot was turned off when only BF measurement was being performed.

Fixation variability was recorded using wearable eye-tracking glasses (Tobii glass II; Tobii Technology, Stockholm, Sweden). These glasses possess a high-definition scene camera with a resolution of 1920 x 1080 pixels, mounted at the centre of the temple, which captures a high-definition video of the view in front of the participant. The glasses also contain eye-tracking sensors with a sampling frequency of 50 Hz, located on the inside of the lower frame, which record the direction of eye gaze. Fixation data recorded with the two cameras were stored in the secure digital card of the recording assistant device. Calibration was performed using the original calibration card, in accordance with the instructions provided in the user manual. To ensure accuracy, calibration was performed and checked immediately before each test. Recalibration was performed before switching measurement eye or if the initial calibration was inaccurate. Recorded data were exported as pixel data for the x- and y-axes and then converted to degrees.

Ocular position and fusional amplitude were respectively measured using a prism bar (Inami, Tokyo, Japan) and major amblyoscope (Haag-Streit UK, Harlow, UK). Near ocular position of 30 cm corrected to far distance was measured with the alternate prism cover test. Fusional amplitude was measured with an over correction of -3.25D added to far correction, because visual field measurement (30 cm) was performed with far correction.

Fixation variability during SAP was measured using calibrated wearable eye-tracking glasses in a single order (DEF, followed by N-DEF, then BF) that was selected at random for the experiment. Because the eye-tracking sensors are contained on the inside of the frame, an occlusion gauze patch was placed over the wearable eye-tracking glasses. The participants were rested for at least 10 min between sessions. Data acquired during the setup and blinking were removed. Fixation variability was expressed as the bivariate contour ellipse area (BCEA), and the amount and frequency of gaze deviations from the fixation target were analysed for each measurement condition during SAP. The relationship of fixation variability with ocular position and fusional amplitude was analysed during BF. Participants with false-positive response rates >15%, false-negative response rates >33%, and fixation-loss rates except in BF >20% were excluded from this study.

For a given proportion of fixation points, BCEA was calculated using the following formula[32]:

$$BCEA=2k*\sigma_{H}*\sigma_{V}*\sqrt{\left(1-\rho^{2}\right)}$$ (1)

where *σ*_*H*_ and *σ*_*V*_ are the standard deviations of the fixation location over the horizontal and vertical meridians, respectively, and *ρ* is the product–moment correlation of these two positional components. The value of k depends upon the area chosen:

$$p=1-e^{-k}$$ (2)

where *e* is the base of the natural logarithm. Therefore, 63.22% of the fixation positions lie within this area when k is 1. For this study, fixation data were calculated with p-values of 0.6827 (k = 1.147), 0.9545 (k = 3.079), and 0.9973 (k = 5.521), corresponding to one, two, and three standard deviations of the fixation location data.

### Statistical analysis

Data were analysed using MedCalc, version 13.2.0.0 (MedCalc Software, Ostend, Belgium), R statistical software (The R Project for Statistical Computing), and G*Power3, version 3.1.7 (Franz Faul, Universität Kiel, Germany).

The paired t-test was used for comparison of mean values between two samples. Bonferroni-corrected probability values of <0.05 were considered to indicate statistically significant differences. Pearson’s correlation coefficient (r) was used for data correlation. The Bonferroni test was used for data comparison at each time point. The effect size, α error, power (1−β error), and non-sphericity correction were 0.25 (middle), 0.05, 0.80, and 0.50, respectively, and the required sample size was 29 participants for three repeated measurements.

## Results

None of the originally enrolled participants were excluded. Table 1 presents the participant demographic characteristics. There were no significant differences in ocular characteristics between dominant and non-dominant eyes. Table 2 presents clinical data measured using the HFA in the DEF, N-DEF, and BF conditions.

**Table 1:** Participant demographic and ocular characteristics.

Parameter	Mean ± SD (range)		p Value
	Dominant Eye	Non-Dominant Eye	
Gender (male/female)	8/27		
Dominant eye (right/left)	26/9		
Age (years)	21.9 ± 1.7 (21 to 29)		
Visual acuity (LogMAR)	-0.28 ± 0.05 (-0.30 to -0.18)	-0.28 ± 0.05 (-0.30 to -0.18)	0.9999
Spherical equivalent (dioptre)	-3.32 ± 2.35 (0.00 to -9.13)	-3.31 ± 2.75 (0.88 to -11.13)	0.6624
Cylindrical power (dioptre)	-0.39 ± 0.41 (0.00 to -1.25)	-0.44 ± 0.40 (0.00 to -1.25)	0.4813
Axial length (mm)	24.67 ± 1.14 (21.40 to 27.00)	24.68 ± 1.17 (21.24 to 26.92)	0.8913
Intraocular pressure (mmHg)	13.6 ± 2.2 (9.0 to 17.3)	13.5 ± 2.3 (8.0 to 19.3)	0.9133

LogMAR, logarithmic minimum angle of resolution; SD, standard deviation.

**Table 2:** Comparison of standard automated perimetry findings among the DEF, N-DEF, and BF conditions.

Parameter	(1) DEF	(2) N-DEF	(3) BF	p Value		
				(1)—(2)	(1)—(3)	(2)—(3)
Mean deviation (dB)	0.70 ± 1.06	0.61 ± 1.03	1.82 ± 1.25	0.8352	< 0.0001	< 0.0001
Visual field Index (%)	99.8 ± 0.4	99.7 ± 0.5	99.7 ± 0.5	0.8305	1	1
Pattern standard deviation (dB)	1.38 ± 0.30	1.41 ± 0.27	1.36 ± 0.30	1	1	1
Foveal threshold (dB)	40.1 ± 1.7	40.1 ± 1.8	41.3 ± 1.7	1	0.0036	0.0014
Test duration (sec)	244.5 ± 19.3	246.3 ± 23.7	249.0 ± 13.1	1	0.6882	1
False positive (%)	0.40 ± 0.91	0.54 ± 1.09	0.37 ± 0.81	0.7715	1	1
False negative (%)	0.06 ± 0.24	0.03 ± 0.17	0.26 ± 0.92	0.9731	0.6844	0.4809
Fixation loss (%)	2.63 ± 4.34	3.02 ± 4.39	NA	0.2008*	NA	NA

p values were adjusted with the Bonferroni correction. DEF, dominant eye fixation; N-DEF, non-dominant eye fixation; BF, binocular fixation.

*calculated with paired t-test.

Table 3 presents the comparison of fixation variability and its average gaze deviations from the fixation target among the DEF, N-DEF, and BF conditions. There were no significant differences among the three conditions in BCEA or average gaze deviations from the fixation target during foveal threshold measurement. During visual field measurement, the BCEA and average gaze deviations from the fixation target in BF were significantly lower than those in DEF (p = 0.0227) and N-DEF (p = 0.0006) conditions. There were no significant differences in BCEA and average gaze deviations from the fixation target between DEF and N-DEF conditions.

**Table 3:** Comparison of fixation variability and average gaze deviations from fixation target among the DEF, N-DEF, and BF conditions.

Parameter	(1) DEF	(2) N-DEF	(3) BF	p Value			
				(1)—(2)		(1)—(3)	(2)—(3)
**Foveal threshold measurement**							
BCEA (deg^2^)							
1SD (68.27%)	1.75 ± 2.73	1.45 ± 1.51	1.67 ± 2.62	0.7624		1	1
2SD (95.45%)	10.29 ± 13.37	8.80 ± 8.89	9.52 ± 14.89	0.8381		1	1
3SD (99.73%)	18.44 ± 23.98	15.77 ± 15.95	17.07 ± 26.69	0.8381		1	1
Average gaze deviations from fixation target (degree)							
	0.92 ± 0.48	0.85 ± 0.38	0.91 ± 0.69	1		1	1
**Visual field measurement**							
BCEA (deg^2^)							
1SD (68.27%)	4.62 ± 3.38	5.24 ± 3.67	2.85 ± 2.00	0.9505		0.0227	0.0006
2SD (95.45%)	27.73 ± 19.84	29.92 ± 19.79	16.78 ± 11.09	1		0.0104	< 0.0001
3SD (99.73%)	49.72 ± 35.58	53.66 ± 35.48	30.10 ± 19.88	1		0.0104	0.0006
Average gaze deviations from fixation target (degree)							
	1.62 ± 0.64	1.69 ± 0.55	1.22 ± 0.46		1	0.0003	0.0001

p values were adjusted with the Bonferroni correction. BCEA, bivariate contour ellipse area; DEF, dominant eye fixation; N-DEF, non-dominant eye fixation; BF, binocular fixation; SD, standard deviation.

**Fig 1:**
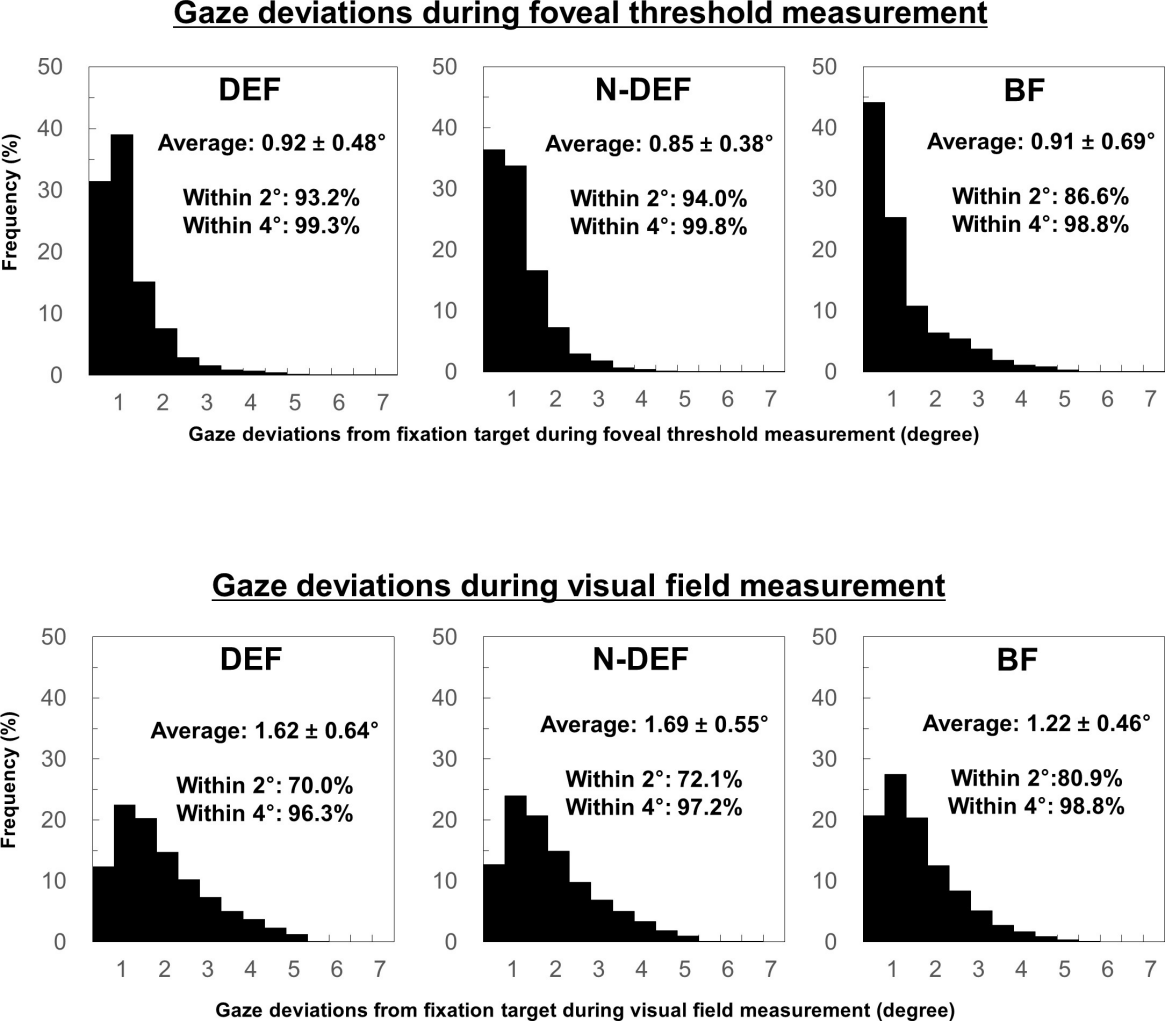
Frequency of gaze deviations from fixation target. Histograms showing the frequency of gaze deviations during foveal threshold (upper) and visual field (lower) measurement in the dominant eye fixation (DEF), non-dominant eye fixation (N-DEF), and binocular fixation (BF) conditions. Data are given as mean ± standard deviation.

Fig 1 presents the frequency of gaze deviations from the fixation target during foveal threshold and visual field measurements. The ranges of average frequency of gaze deviations within 2° and 4° from the fixation target among each fixation condition were 86.6–94% and 98.8–99.8%, respectively, during foveal threshold measurement; the corresponding values were 70.0–80.9% and 96.3–98.8%, respectively, during visual field measurement.

**Fig 2:**
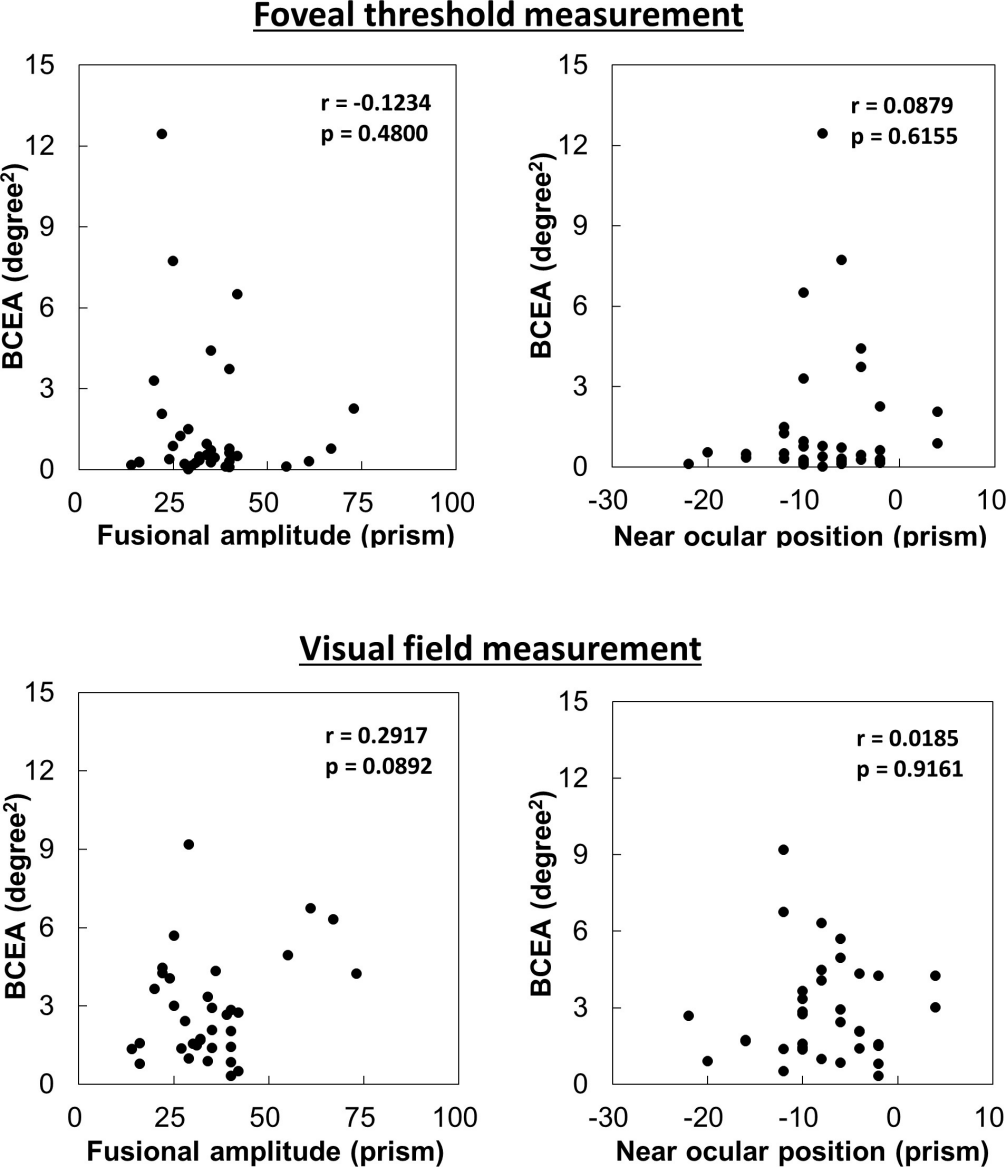
Association of fixation variability in binocular fixation with fusional amplitude and near ocular position. Scatter plots show the correlation of fixation variability during foveal threshold (upper) and visual field (lower) measurement in binocular fixation with fusional amplitude and near ocular position. BCEA, bivariate contour ellipse area.

Near ocular position and fusional amplitude were -7.5 ± 6.5 prism and 38.6 ± 15.1 prism, respectively. There was no significant correlation between BCEA and ocular position or fusional amplitude during foveal threshold or visual field measurements (r = 0.0879–0.2917). Scatter plots for these relationships are presented in Fig 2.

**Fig 3:**
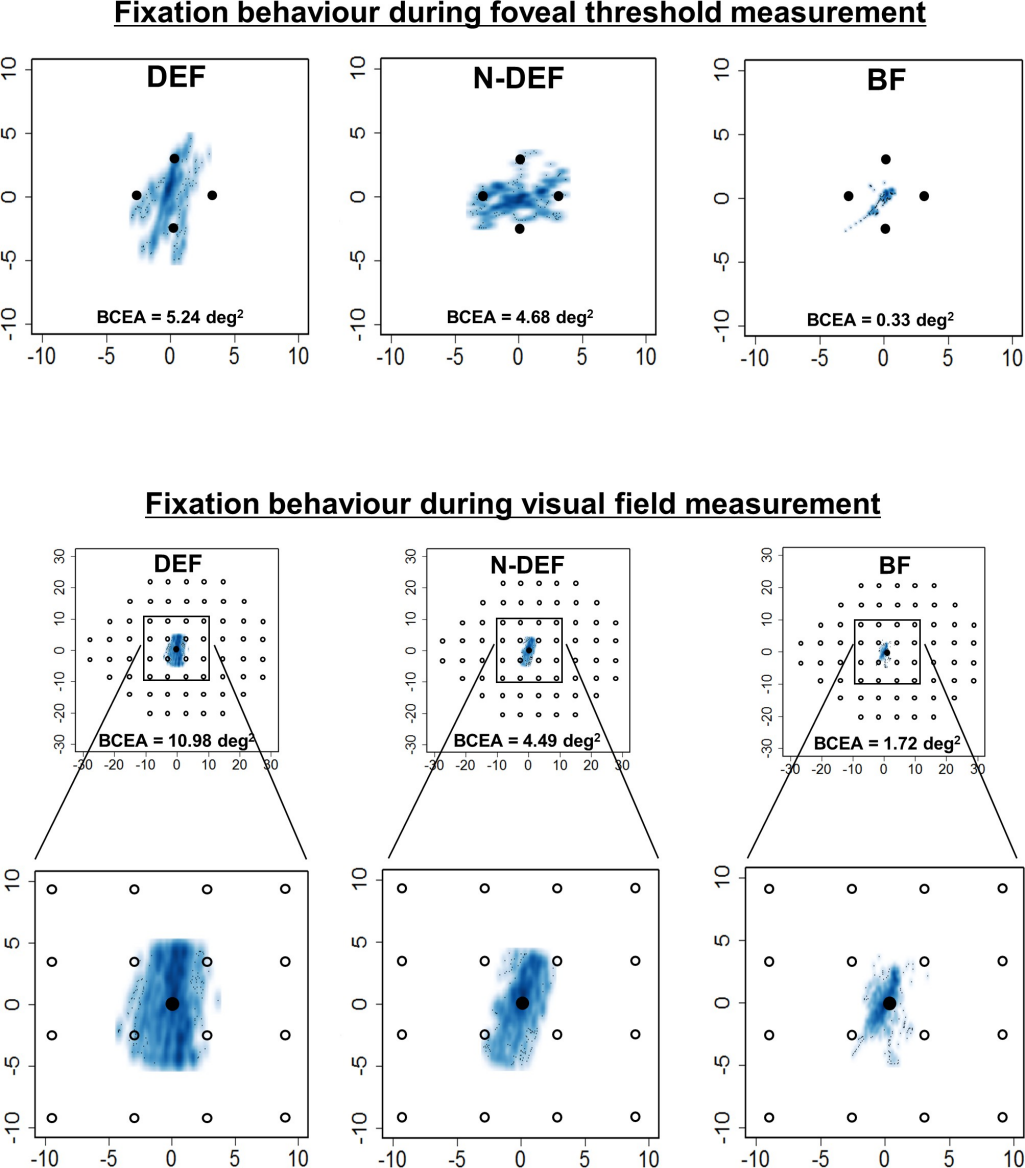
Typical examples of fixation variability. Smooth scatter plots show typical examples of fixation variability during foveal threshold (upper) and visual field (lower) measurement in dominant eye fixation (DEF), non-dominant eye fixation (N-DEF), and binocular fixation (BF) conditions. Fixation variability of the visual field within 30° (upper) and 10° (magnified; lower) in visual field measurement. BCEA, bivariate contour ellipse area.

Fig 3 presents typical examples of fixation variability during foveal threshold and visual field measurements.

## Discussion

In the present study, we used SAP to compare fixation variability in the DEF, N-DEF, and BF conditions and evaluated the association of fixation variability with ocular position and fusional amplitude during BF. There was no significant difference in fixation variability between DEF and N-DEF conditions. However, fixation in BF was improved relative to fixation in the monocular condition. During visual field measurement, the ranges of average frequency of gaze deviations within 2° and 4° from the fixation target were 70.0–80.9% and 86.6–94%, respectively. In addition, there was no correlation between fixation variability in BF and ocular position or fusional amplitude.

Previous comparisons of functional measurements during SAP have demonstrated no significant differences in retinal sensitivity, [33] accommodation power,[34] or reading speed[35] between DEF and N-DEF conditions. Our present study also found no significant differences in HFA parameters between DEF and N-DEF conditions.

Although there was no difference in fixation variability among the fixation conditions during foveal threshold measurement, fixation variability during visual field measurement was lower in BF than in DEF and N-DEF. For the HFA, which consists of a bowl of 30-cm radius, accommodation and convergence were induced during SAP. A previous study involving participants with exophoria reported lower accommodation lag in BF than in monocular fixation.[36] Participants in the present study mostly exhibited the ortho or phoria eye position, without heterotropia. Therefore, fixation variability in BF might have been expected to be more stable than fixation variability in the monocular condition. However, it appears that the results could vary because BF is difficult to achieve in elderly subjects with weak accommodation, anisometropia, strabismus, convergence insufficiency, or low fusional amplitude.

Fixation variability in BF was not correlated with fusional amplitude or near ocular position. This is because the present study only included healthy young participants in their early twenties, who had adequate fusional amplitude and accommodative power and could, therefore, stably fixate on the fixation target at a distance of 30 cm. The fixation variability in BF is thought to be unstable in elderly people with declined accommodation power, patients with narrow fusional amplitude, and patients with glaucoma or other eye diseases who possess different degrees of visual field abnormalities in each eye. Further investigation is needed to evaluate this trend in a wide range of participants.

Previous studies that evaluated fixation variability using fundus-tracking perimetry had reported BCEA values of 0.61 and 1.16 logBCEA in 29 healthy participants[19] and 4.79 deg^2^ in patients with low vision.[26] In the present study, the logBCEA and BCEA values were 0.35–0.61 and 2.85–5.24 deg^2^, respectively. In a previous study that employed fundus-tracking perimetry, the frequencies of gaze deviations within 2° and 4° from the fixation target were 86% and 96%, respectively, in healthy participants.[37] In the present study, the frequencies of gaze deviations within 2° and 4° from the fixation target were 70–80.9% and 96.3–98.8%. Fixation variability in the present study was slightly higher than that reported in previous studies, which might be related to differences in tracking system (pupil- and fundus-tracking), sampling rate, and test duration between the present study and previous studies.

The present study has some limitations. First, participants included in this study were all healthy young subjects. Because fixation variability during static perimetry is relatively high in patients with ocular disease,[10, 14, 18, 20–23, 25, 27] the present results should be also verified in patients with glaucoma and other ocular diseases. Second, because of the pupil-tracking approach, it was not possible to detect rotatory deviation using the wearable eye-tracking glasses and the slightest movement of the wearable eye-tracking glasses during measurement could also produce an impression of fixation variability, even in the absence of actual fixation variability. Third, although the present sample size was adequate for repeated-measures analysis of variance among the three groups, it was inadequate for correlation coefficient analysis. Forth, we were not able to distinguish between genuine fixation wondering and gaze attraction from projected stimuli during the perimetric test. Fifth, ten minutes interval between each test might be insufficient to exclude a fatigue effect. Therefore, further studies should be undertaken to evaluate the association of fixation variability with ocular position and fusional amplitude during BF.

In conclusion, there was no significant difference in fixation variability between monocular and binocular fixation in a short-duration foveal threshold measurement test. However, in a long-duration test, such as visual field measurement, fixation variability in BF might be reduced.

## Supporting information

S1 File The data analysed in the current study. (XLSX)
